# The moderating role of mental health on the association between gaming time and gaming disorder symtoms

**DOI:** 10.1192/j.eurpsy.2022.246

**Published:** 2022-09-01

**Authors:** P. Koncz, Z. Demetrovics, B. Paksi, A. Magi, A. Eisinger, O. Király

**Affiliations:** 1 Eötvös Loránd University, Institute Of Psychology, Budapest, Hungary; 2 Eötvös Loránd University, Doctoral School Of Psychology, Budapest, Hungary; 3 University of Gibraltar, Centre Of Excellence In Responsible Gaming, Gibraltar, Gibraltar; 4 ELTE Eötvös Loránd University, Institute Of Education, Budapest, Hungary

**Keywords:** Gaming Disorder, escapism, adhd, Depression

## Abstract

**Introduction:**

Video games are among the most popular leisure time activities. While majority of gamers play in a healthy manner, a minority shows gaming disorder (GD) symptoms and experiences detrimenting effects in their lives. Even though gaming time is moderately associated with gaming disorder symtoms, research suggests that it is not a reliable predictor by itself.

**Objectives:**

The aim of the present study is to explore whether depression symptoms, self-esteem, attention-deficit/hyperactivity disorder (ADHD) and escapism (when gaming is motivated by the avoidance of everyday problems) moderate the association between gaming time and GD symptoms and whether this is different for boys and girls.

**Methods:**

Data was collected from a representative sample of 5^th^ grade students of public education institutions in Budapest. Sample selection was carried out by one-step sampling stratified for school type, district, and maintainer; the sampling unit was the class. Data from 2126 students were analyzed (49.3% male, mean age 10.7 years, SD=0.54).

**Results:**

Depression symptoms moderated the association between gaming time and GD symtoms in both genders. For those with higher depression symptoms the aforementioned association was stronger. Furthermore, self-esteem had a moderator effect only among girls, while escapism motivation and ADHD only among boys. In these cases, the association between gaming time and GD symptoms was stronger among those with lower self-esteem, and higher ADHD and escapism scores.

**Conclusions:**

Results suggest that gaming time is more strongly connected to GD symptoms in certain conditions, but its predictive value is limited even in those cases.

**Disclosure:**

Funding statement: This study was supported by the Hungarian National Research, Development and Innovation Office (KKP126835). Orsolya Király was supported by the János Bolyai Research Scholarship of the Hungarian Academy of Sciences and by the ÚNKP-21-5

